# Gait Trajectory and Gait Phase Prediction Based on an LSTM Network

**DOI:** 10.3390/s20247127

**Published:** 2020-12-12

**Authors:** Binbin Su, Elena M. Gutierrez-Farewik

**Affiliations:** 1 KTH MoveAbility Lab, Department of Engineering Mechanics, Royal Institute of Technology, 10044 Stockholm, Sweden; binbins@kth.se; 2 KTH BioMEx Center, Royal Institute of Technology, 10044 Stockholm, Sweden; 3 Department of Women’s and Children’s Health, Karolinska Institutet, 17177 Stockholm, Sweden

**Keywords:** multi-step forecasting, gait segmentation, lower limb angular velocity, machine learning, deep learning

## Abstract

Lower body segment trajectory and gait phase prediction is crucial for the control of assistance-as-needed robotic devices, such as exoskeletons. In order for a powered exoskeleton with phase-based control to determine and provide proper assistance to the wearer during gait, we propose an approach to predict segment trajectories up to 200 ms ahead (angular velocity of the thigh, shank and foot segments) and five gait phases (loading response, mid-stance, terminal stance, preswing and swing), based on collected data from inertial measurement units placed on the thighs, shanks, and feet. The approach we propose is a long-short term memory (LSTM)-based network, a modified version of recurrent neural networks, which can learn order dependence in sequence prediction problems. The algorithm proposed has a weighted discount loss function that places more weight in predicting the next three to five time frames but also contributes to an overall prediction performance for up to 10 time frames. The LSTM model was designed to learn lower limb segment trajectories using training samples and was tested for generalization across participants. All predicted trajectories were strongly correlated with the measured trajectories, with correlation coefficients greater than 0.98. The proposed LSTM approach can also accurately predict the five gait phases, particularly swing phase with 95% accuracy in inter-subject implementation. The ability of the LSTM network to predict future gait trajectories and gait phases can be applied in designing exoskeleton controllers that can better compensate for system delays to smooth the transition between gait phases.

## 1. Introduction

Lower limb exoskeletons (LLEs) have for decades received increasing attention and interest in the field of rehabilitation for patients with a disability affecting their ability to locomote bipedally. They can provide vertical support, assistance in lower limb motion, and propulsion of the body to help wearers regain locomotion function. Trajectory-tracking controllers are widely adopted in such exoskeletons to create repetitive motion patterns [1]. Assistive exoskeletons can guide wearers’ movement in a predefined trajectory by exerting the necessary torque around the joints. Thus, a vital function for controlling LLEs during walking is to generate a gait trajectory, i.e., movement of the lower limb joints and segments. If a gait trajectory can be predicted and incorporated into the control algorithm, it may be beneficial to add a feed-forward component to compensate for the delay that may result in the control’s response time [2,3,4]. Both model-based and machine learning methods are widely studied approaches for gait trajectory prediction.

Model-based optimization methods are popular in predicting gait trajectory through biomechanical models, for example, Hill-type muscle models, computerized musculoskeletal models [5], and in computing cost functions under certain constrains, such as joint angle limits, joint torque limits and unilateral contact constraints [6,7]. The reliability and applicability of this approach depend on how biofidelic the model is and on how well the cost function represents a person’s movement strategy. Errors in the model’s kinetic response will affect the predicted muscle loads and thus the calculated metabolic cost. More realistic and individualized models inevitably impose heavier burdens on the computational cost than generic scaled models. Furthermore, while the choice of cost function affects simulation results significantly, its formulation still remains a topic of ongoing scientific debate [8], particularly for persons with a gait pathology.

Machine learning methods have gradually become a viable approach for gait trajectory prediction, as such methods are based on a large amount of data, and are free from biomechanical models and cost functions [9]. Studies have estimated gait trajectories based on body parameters such as age, height and mass [10] and the targeted walking speed [11]. Several studies consider gait trajectory as a form of time series in which the values change over time. As such, gait trajectory prediction is essentially a time series prediction in which a sequence of future values are predicted based on a sequence of past observations [9,12,13]. Long-short term memory (LSTM) is reported to be one of the most effective models to deal with sequential data, initially proposed by Hochreiter and Schmidhuber, who introduced self-loops to generate paths where the gradient can flow for long durations [14]. There are three major components in an LSTM network: the input gate, the forget gate and the output gate. The input gate decides how much information to add to the cell state. The forget gate determines how much information from the previous cell state to forget. The output gate controls the information to output based on the cell state. LSTM performs especially well for noisy and non-stationary time series prediction and classification, and has been shown to be well-suited to target human gait classification [15], time series classification from accelerometer-based data [16], adaptive trajectory generation of lower limb exoskeletons for stroke rehabilitation [17] and intent prediction from motion trajectories [18]. Liu et al. [19] used a Deep Spatial-Temporal Model to generate a knee joint trajectory one time frame in advance based on the historic gait trajectory of other joints in able-bodied subjects, and applied the new trajectories on a lower-limb exoskeleton for subjects with knee injury. Zaroug et al. [20] used LSTM autoencoders to forecast trajectories of the lower limb kinematics, specifically linear acceleration and angular velocity (AV), and report a correlation coefficient between measured and predicted trajectories of 0.98. Moreira et al. [21] implemented LSTM models to generate healthy reference ankle joint torques of subjects walking on a flat surface, achieving a normalized Root Mean Square Error of 4.31%, showing that the LSTM has the potential to be integrated into control architectures of robotic assistive devices to accurately estimate healthy user-oriented reference ankle joint torques. Despite the progress made thus far in predicting gait trajectory, most previous studies have focused on predicting gait trajectory for the next single time frame, and in which the prediction step is recurrently used as input for the next prediction. One major shortcoming of such an approach is that errors occurring at some steps tend to propagate and magnify in subsequent prediction, potentially resulting in poor accuracy. Multiple-frame prediction can allow for a slower update rate of the model predictive control and can maintain smoothness and accuracy of the resulting control system response [22], neither of which has previously been explicitly discussed and addressed in gait trajectory prediction.

Another critical factor in LLE control is the gait phase. When a patient wears an exoskeleton, a gait phase-modulated torque is commonly provided to assist the patient. Martini et al. [23] provided hip flexor assistance during swing to reduce the total energy cost of walking with a powered hip exoskeleton. Kazerooni et al. [24] divided an entire gait cycle into a loaded stance phase and an unloaded swing phase in the hybrid control of the BLEEX exoskeleton and used position control in stance and positive feedback control in swing. Accurate identification of gait phases is crucial not only for metabolically-efficient LLE control but also to prevent falsely exerted joint torque from inaccurately detected gait phases [25]. In addition, a discrete-phase-control LLE can initiate movements before the actual gait phase to make the transition smoother. Research has been conducted extensively in gait phase detection based on the current gait phase, using a range of wearable sensors including foot switches, pressure insoles, accelerometers, gyroscopes, and IMUs [26,27,28]. Multiple-frame gait phase prediction implies earlier prediction of a user’s motion intention, which would be useful in addressing both lags in the information measured by mechanical sensors on the exoskeleton or user, and in delays involved in controlling a motor. While a few studies have predicted the gait phase in the next time frame [29,30,31], gait phase prediction for multiple upcoming time frames has otherwise been little investigated.

The objectives of this study are to develop an approach for multiple-frame prediction of gait trajectory and gait phases based on a long short-term memory (LSTM) network in which a discount factor in the loss function is introduced. This approach involves a model that maps the multiple-frame output directly with past values to avoid iterative error accumulation and a discount factor that is customized to more accurately predict up to 10 time frames in the near future, equivalent to 200 ms at the proposed sensor frequency, while maintaining an overall good performance during the entire prediction horizon. To the best of the authors’ knowledge, a discount factor has not yet been applied in gait trajectory prediction.

## 2. Materials and Methods

### 2.1. Data Collection and Processing

A convenience sample of 12 able-bodied subjects (6 males and 6 females) between 25 and 30 years old were recruited among students and colleagues. Each subject was asked to walk on a treadmill at five different speeds that correspond to mean, mean ±1 standard deviation (SD), and mean ±2 SDs walking speed, based on reported comfortable walking speeds normalized to subjects’ ages and height [32]. Data were collected for 300 s for each walking speed, for a total of between 1500 and 1700 gait cycles per person. The order of the speeds was randomized in data collection.

Subjects were equipped with seven IMUs (Myon/Cometa aktos-T), attached with tape to the thighs, shanks, feet and pelvis. They were also equipped on each foot with 4 foot switches, small piezoresistive pressure sensors taped to the soles of the feet at the heels, first and fifth metatarsal heads and big toe, that record an on or off signal based on ground contact. Data were collected at 2000 Hz and down-sampled to 50 Hz to reduce computational load. Data collection for this study was approved by the Swedish Ethical Review Authority (Dnr. 2020-02311). All subjects gave informed written consent to participate. Participation was voluntary and could be discontinued at any time.

The labeling procedure of each gait phase was implemented by defining the phases detected by foot switches as the “ground truth” for 5 gait phases. Four phases during stance were defined as per Gage et al. [33]: loading response (LR), midstance (MS), terminal stance (TS), pre-swing (PSw), based on the on/off signal of each foot switch, signaling contact with the ground. LR begins at ipsilateral foot contact and ends at contralateral foot-off, followed by MS, which ends at ipsilateral heel off, followed by TS, which ends at contralateral foot contact, and finally followed by PSw, which ends at ipsilateral foot off [34]. Since foot switches provide no information during Swing (Sw), the Sw phase was defined as the entire duration of swing, i.e., not defined into any subphases.

Collected IMU data were normalized to mean zero and unit variance using z-score normalization. The size of IMU data is M×N where *M* represents the total number of recorded samples and *N* represents the number of feature variables, namely, 63 variables from the 7 IMUs and 9 channels (acceleration, angular velocity and magnetic field intensity in 3 directions) per IMU. The gait trajectories, defined here as the angular velocities of thigh, shank and foot, were acquired directly from the IMU sensors. The foot switches were solely used for labeling each IMU data into corresponding gait phase and were not used as inputs to the LSTM network. Before being fed into the LSTM model, the IMU data were transformed into a 3D dataset using a sliding window technique [35]. The sliding window consisted of an input window, an output window and a sliding size. The input window consists of *P* samples and *N* features, and the output window comprises of *F* samples and *N* features where *P* and *F* depends on the time span of past observation and future prediction. The input window is the input data to the LSTM model, and the output window is the future prediction output from the LSTM model. The input window size was chosen to be 10 and 30, respectively, for 5 and 10 time steps prediction (output window), equivalent to a 100 and 200 ms ahead prediction based on the grid search approach. The sliding size is how much of *M* samples that both the input and the output windows are sliding forward with. The sliding size was one.

### 2.2. LSTM Model for Gait Trajectory and Phase Prediction

**Figure 1:**
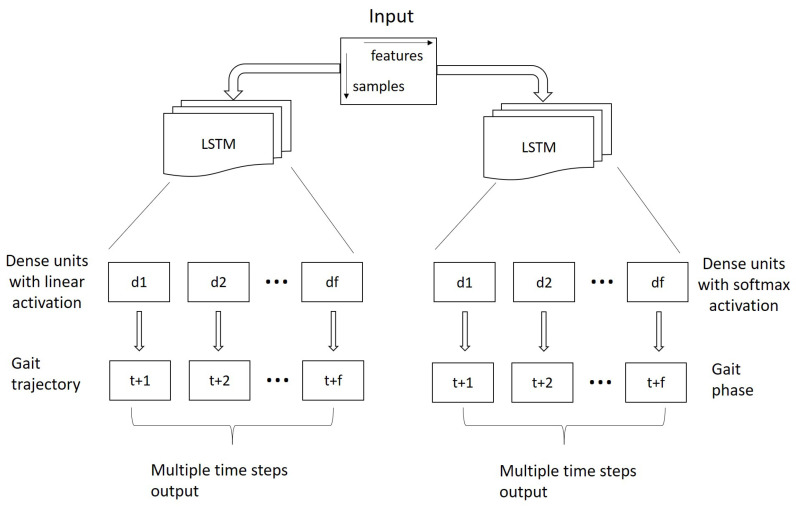
Implemented long-short term memory (LSTM) architecture for multiple-frame output of gait trajectory and phases.

The LSTM model for gait trajectory and phase prediction is illustrated in Figure 1. The input of the LSTM has a dimension of P×N where *P* represents the number of samples from the past and *N* represents the number of feature variables, namely, 63 variables from the 7 IMUs and 9 channels (acceleration, angular velocity and magnetic field intensity in 3 directions) each IMU. The number of LSTM units depends on the number of future steps to predict. We set the number of LSTM units to 5 times the future time frames in order to create enough capacity for the model. The number of dense layers is equal to the number of future time frames to predict which one matches the multiple-frame output.

For gait trajectory prediction, each dense layer has a linear activation. The network weights and biases were updated at the end of each batch using an adaptive moment estimation (Adam) optimization algorithm with discounted mean square error (MSE) and a regularization term (Equation (2)) as an optimization criterion.

For gait phase prediction, each dense layer has a softmax activation. The network weights and biases were updated at the end of each batch using an adaptive moment estimation (Adam) optimization algorithm with discounted categorical cross-entropy and a regularization term (Equation (2)) as an optimization criterion.

### 2.3. Multiple-Frame Gait Trajectory or Gait Phase Predictions

The IMU signals *X* and prediction of gait trajectories or gait phases *Y* of *T* frames can be expressed as $X=\{x_{1},x_{2},x_{3},\ldots ,x_{t},\ldots ,x_{T}\}$ and $Y=\{y_{1},y_{2},y_{3},\ldots ,y_{t},\ldots ,yT\}$. We used the notation $X_{t-p+1}^{t}$ and $Y_{t+1}^{t+f}$ to denote a sequence of p−1 past IMU signals and *f* future prediction at current time *t* where $X_{t-p+1}^{t}=\left\{x_{t-p+1},x_{t-p+2},\ldots ,x_{t}\right\}$ and $Y_{t+1}^{t+f}=\left\{y_{t+1},y_{t+2},\ldots ,y_{t+f}\right\}$. The task was to predict a sequence of *f* future values of lower segment trajectories or gait phases $Y_{t+1}^{t+f}$ given *p* past observation of IMU signals $X_{t-p+1}^{t}$. The relationship between the past and future values can be represented by the following form:

(1) $$Y_{t+1}^{t+f}=F_{\theta }\left(X_{t-p+1}^{t}\right)+\epsilon_{i}$$

where *F* is a function that matches the past observation and future prediction parameterized by θ, and $\epsilon_{i}$ is the noise, which compensates for the difference between outputs of the function and real target values with zero mean and variance $\sigma^{2}$ that corresponds to each future frame.

If many frames are to be predicted into the future, it is often practical to assume that the immediate frames are easier to predict than the more distant ones. Therefore, it would be preferable to make predictions that are weighted differently based on their immediacy. If {${\hat{y}}_{t+1}$, ${\hat{y}}_{t+2}$, …, ${\hat{y}}_{t+f}$} denotes the estimated values from the function $f_{\theta }$, we propose to minimize a designated loss function to determine the optimal parameter $\theta^{*}$.

(2) $$\theta^{*}=arg\min_{\theta }\sum_{0}^{f-1}\alpha^{i}{\left[y_{t+1+i}-f_{\theta }\left(X_{t-p+1}^{t}\right)\right]}^{2}+\lambda \sum \left|\theta \right|$$

where α is a discount factor (0≤α≤1) to model the uncertainty in the future, to force the function to focus more on predicting the immediate time frames, λ∑|θ| is a penalty that causes a subset of parameters to become zero thus simplifying the function. With such a loss function, the loss value is the weighted sum of all individual losses of each output, weighted by the coefficients generated from the discount factor and the weight of individual losses falls exponentially with future steps, which means it puts decreasing weight on increasingly distant events. By assigning less weight to the outputs in the more distant future, the model parameter could be retroactively updated to make a more accurate prediction on the most immediate time frames. Although this approach cannot guarantee finding the global minimum for the loss function, it can help reach a local minimum faster, which is often close to the global minimum in neural networks.

### 2.4. LSTM Performance Evaluation

The LSTM was implemented 2 different ways.

Intra-subject: pooled data for each subject and speed, then randomly trained on 70% data and tested on the remaining 30% data for each subject individually.

Inter-subject: pooled data of all other speed and subjects except one (leave-one-out), and tested on the remaining subject, iterated for each subject.

To evaluate the performance of the LSTM model, parameters were computed to compare how accurate the gait trajectory and phases prediction was in each implementation. For gait phase prediction, accuracy was computed for each implementation as the proportion of correctly classified phases, i.e., classified phases that matched the true phases across *f* future steps of *n* samples (Equation (3)). For gait trajectory prediction, defined here as the angular velocity of the thigh, shank and foot of the ipsilateral side in the sagittal plane, the error (Equation (4)), mean absolute error (MAE, Equation (5)) and root mean squared error (RMSE, Equation (6)) were computed across *f* future steps of *n* samples.

(3) $$A_{cc}=\frac{1}{nf}\sum_{j=1}^{n}\sum_{i=0}^{f-1}b\left\{\begin{array}{cc} b=1, & \mathrm{if}\;y_{t+1+i}^{j}={\hat{y}}_{t+1+i}^{j}\\ b=0, & \mathrm{Otherwise} \end{array}\right.$$

(4) $$E_{rr}=\frac{1}{nf}\sum_{j=1}^{n}\sum_{i=0}^{f-1}y_{t+1+i}^{j}-{\hat{y}}_{t+1+i}^{j}$$

(5) $$E_{MA}=\frac{1}{nf}\sum_{j=1}^{n}\sum_{i=0}^{f-1}\left|y_{t+1+i}^{j}-{\hat{y}}_{t+1+i}^{j}\right|$$

(6) $$E_{RMS}=\frac{1}{nf}\sum_{j=1}^{n}\sum_{i=0}^{f-1}{\left(y_{t+1+i}^{j}-{\hat{y}}_{t+1+i}^{j}\right)}^{2}$$

The Pearson correlation coefficient (*R*) was calculated between the known measured trajectories and the predicted output as Equation (7):

(7) $$R\left(y,\hat{y}\right)=\frac{cov(y,\hat{y})}{\sigma_{y}\sigma_{\hat{y}}}$$

where $cov(y,\hat{y})$ is the covariance between actual measured and estimated trajectories. $\sigma_{y}$ and $\sigma_{\hat{y}}$ are the standard deviations of *y* and $\hat{y}$, respectively. The coefficient *R* returns a value between −1 and 1, which represents the limits of correlation from a full negative correlation to a full positive correlation. A value of 0 indicates no correlation.

The signal to noise ratio (SNR) was calculated according to [36] as Equation (8):

(8) $$R_{SN}\left(y,\hat{y}\right)=10\log_{10}\frac{Var(y)}{E_{MS}\left(y,\hat{y}\right)}$$

where Var(y) is the variance of the actual measured trajectories, and $E_{MS}\left(y,\hat{y}\right)$ is the mean squared error between actual measured and estimated trajectories. The ratio between Var(y) and $E_{MS}\left(y,\hat{y}\right)$ was converted into a decibel (dB) scale. SNR=0 indicates that the signal and the noise are equally present in the reconstructed kinematic parameter. SNR<0 (poor prediction) indicates a noisy reconstruction, while SNR>0 (good prediction) indicates a high-quality reconstruction of the signal [37].

## 3. Results

**Figure 2:**
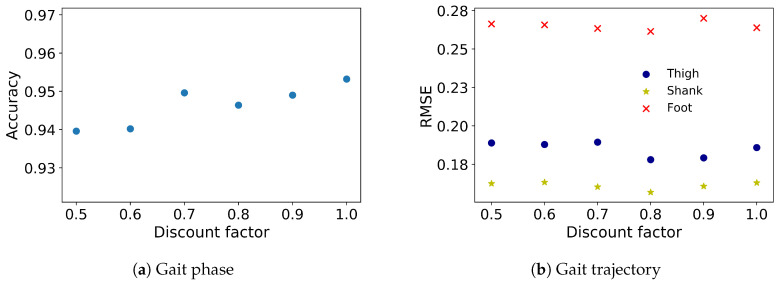
(**a**) Accuracy for gait phases prediction and (**b**) root mean squared error (RMSE) for z-score normalized angular velocities of thigh, shank and foot in 100 ms ahead prediction of one representative subject with respect to different discount factors.

**Figure 3:**
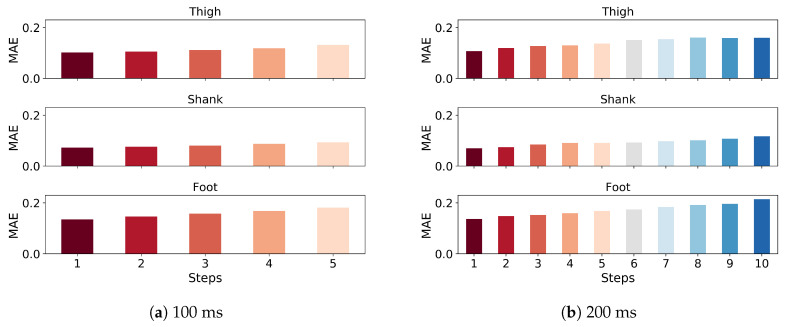
Mean absolute error (MAE) for z-score normalized angular velocities of thigh, shank and foot of one representative subject in 100 ms (**a**) and 200 ms (**b**) ahead prediction. Each step repesents 20 ms.

The LSTM architecture, coded in Python 2, was trained and tested on a laptop computer (Thinkpad T470p) with an Intel i7-7700HQ CPU @ 2.8 GHz and 8 GB RAM. The model’s hyperparameters were determined with the grid search approach, including number of epochs, batch size, layers and cells. The optimum model was then trained for 100 epochs with an early stop if the model performance did not increase in the consecutive 10 epochs, and performance was evaluated on the test set using accuracy and RMSE for gait phase prediction and gait trajectory prediction, respectively. The results of 5 and 10 time frames (100 and 200 ms, respectively, at 50 Hz) are shown in different implementations separately. The discount factor was varied from 0.5 to 1, where 1 is no penalty, to compare the RMSEs for angular velocities of thigh, shank and foot (100 ms ahead prediction) of one representative subject and the accuracy for gait phases (Figure 2). The lowest RMSE and the highest accuracy occurred in a discount factor of 0.8 and 1, respectively. The MAE between the true and predicted trajectory of angular velocity in 100 and 200 ms prediction indicates better performance in the earlier steps (Figure 3). While the MAE error gradually increases as more time steps are introduced, the increments are negligible and the error is overall low. The angular velocity of the shank has the lowest prediction error.

### 3.1. Intra-Subject Implementation

For each subject, we pooled the data for all speeds and iterated over the training and validation data selection.

**Figure 4:**
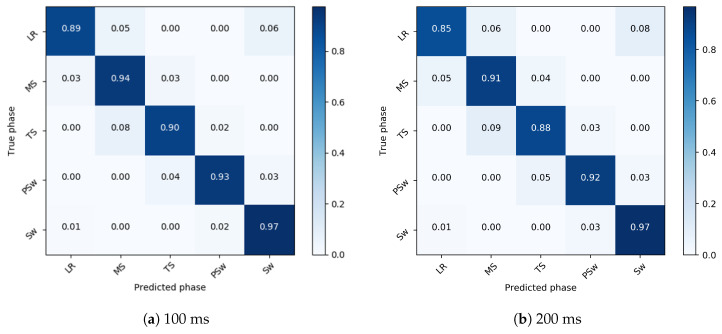
Confusion matrix showing the gait phase prediction accuracy of 100 ms (**a**) and 200 ms (**b**) ahead in the intra-subject implementation. The true gait phase is shown on the *y*-axis and the predicted phase is on the *x*-axis. A strong diagonal indicates very accurate prediction, and misclassifications are indicated off the diagonal.

In gait phase prediction 100 ms ahead, the model most accurately detected Sw (97%), followed by MS (94%), PSw (93%), TS (90%), and LR (89%), for an overall accuracy of 94%. Recognition variability was highest in LR and TS (Figure 4). In predictions 200 ms ahead, the model most accurately detected Sw (97%), followed by PSw (92%), MS (91%), TS (88%), and LR (85%), for an overall accuracy of 92%. Recognition variability was also highest in LR and TS in the 200ms ahead prediction.

**Figure 5:**
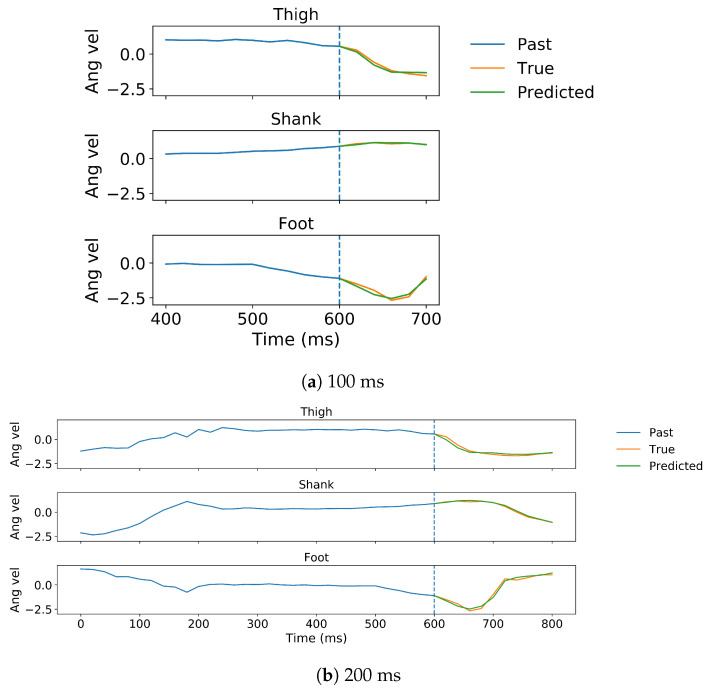
The 100 ms (**a**) and 200 ms (**b**) ahead predictions for z-score normalized angular velocities of ipsilateral thigh, shank and foot of one representative subject in the intra-subject implementation, shown for prediction from the same point in time. All angular velocity values shown are normalized to have zero mean and unit variance. The predicted trajectory is shown in green and the true in orange. Note that more past data are used for the 200 ms ahead prediction than for the 100 ms ahead prediction.

**Figure 6:**
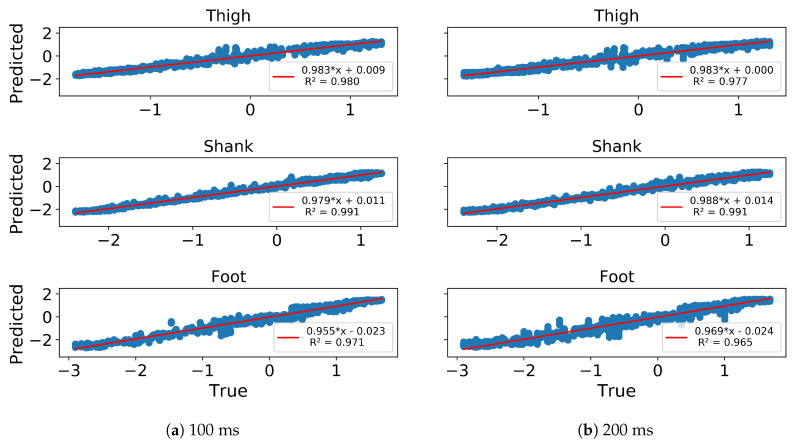
Linear correlation of Predicted (*y*-axis) vs. True (*x*-axis) z-score normalized angular velocities of ipsilateral thigh, shank and foot of a representative subject in 100 ms (**a**) and 200 ms (**b**) ahead prediction. All angular velocity values shown are normalized to have zero mean and unit variance.

**Figure 7:**
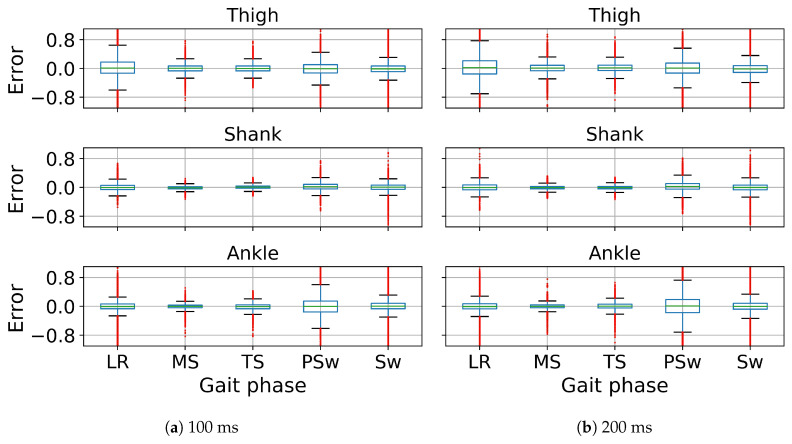
Error of z-score normalized angular velocities of ipsilateral thigh, shank and foot segments in 100 ms (**a**) and 200 ms (**b**) ahead prediction with respect to gait phases in intra-subject implementation. All angular velocity values shown are normalized to have zero mean and unit variance. The line inside the box represents the median error. The box represents the middle 50% of the error. The whiskers represent the ranges for the bottom 25% and the top 25% of the error, excluding outliers. The red tails indicate the outliers.

In gait trajectory prediction, examples of predicted and true values of angular velocity of thigh, shank and foot segments in the 100 and 200 ms ahead predictions are illustrated (Figure 5). The model overall accurately predicted the angular velocities of the lower limb segments. Correlation between predicted and true values for the angular velocity of the thigh, shank and the foot indicate there is a strong relationship between the predicted and true trajectory (illustrated for one subject in Figure 6). The RMSE and MAE were the lowest while the R and SNR were the highest in the shank both in 100 and 200 ms ahead prediction (Table 1). The errors were similar in both implementations but the variance is generally lower in the 100 ms than in the 200 ms ahead prediction. The median error of the gait trajectory with respect to each gait phase was always close to zero but the spread of error varied among phases. The errors had the smallest spread in MS and TS. The median and the spread of error of the shank trajectory was smaller than the thigh or foot trajectories (Figure 7).

### 3.2. Inter-Subject Implementation

**Figure 8:**
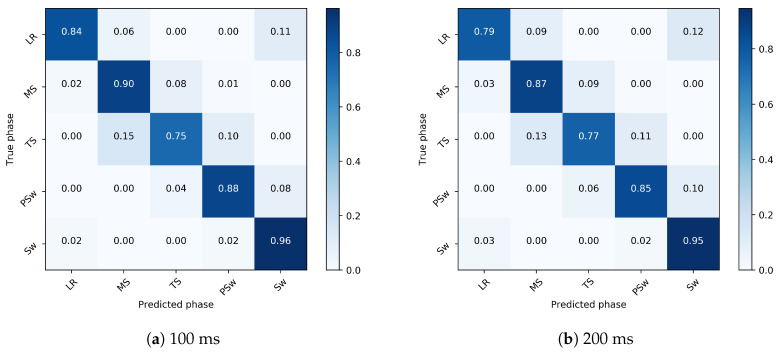
Confusion matrix showing the gait phase prediction accuracy of 100 ms (**a**) and 200 ms (**b**) ahead in the inter-subject implementation. The true gait phase is shown on the *y*-axis and the predicted phase is on the *x*-axis. A strong diagonal indicates a very accurate prediction, and misclassifications are indicated off the diagonal.

In gait phase prediction in the 100 ms ahead prediction, the model most accurately detected Sw (96%), followed by MS (90%), PSw (88%), LR (84%), and TS (75%), for an overall accuracy of 88.7% in 100 ms ahead prediction. Recognition variability was highest in LR and TS (Figure 8). In the 200 ms ahead prediction, the model most accurately detected Sw (95%), followed by MS (87%), PSw (85%), LR (79%), and TS (77%), for an overall accuracy of 86.3%. Recognition variability was again highest in LR and TS.

**Figure 9:**
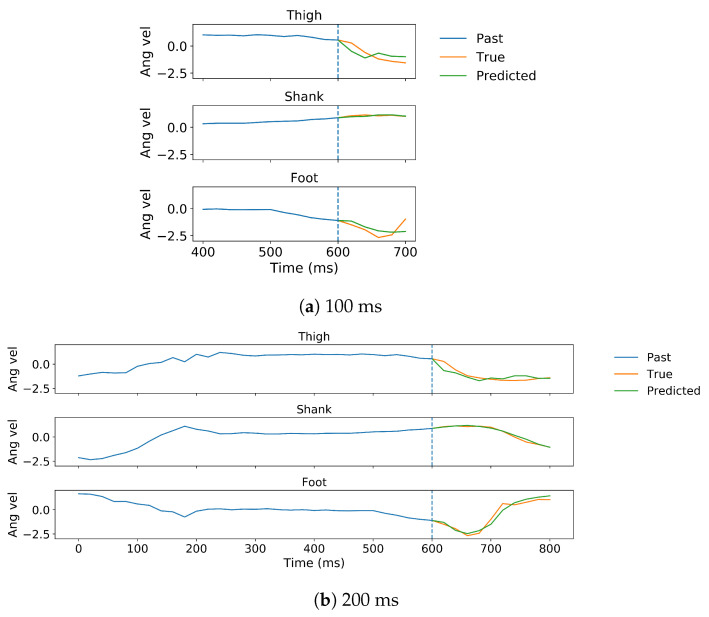
The 100 ms (**a**) and 200 ms (**b**) ahead predictions for z-score normalized angular velocities of ipsilateral thigh, shank and foot of one representative subject in the inter-subject implementation, shown for prediction from the same point in time. All angular velocity values shown are normalized to have zero mean and unit variance. The predicted trajectory is shown in green and the true in orange. Note that more past data are used for the 200 ms ahead prediction than for the 100 ms ahead prediction.

**Figure 10:**
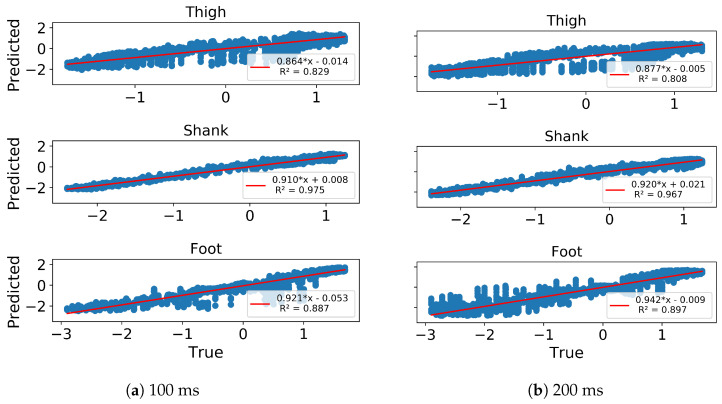
Linear correlation of Predicted (*y*-axis) vs. True (*x*-axis) z-score normalized angular velocities of ipsilateral thigh, shank and foot of a representative subject in 100 ms (**a**) and 200 ms (**b**) ahead prediction in inter-subject implementation. All angular velocity values shown are normalized to have zero mean and unit variance.

**Figure 11:**
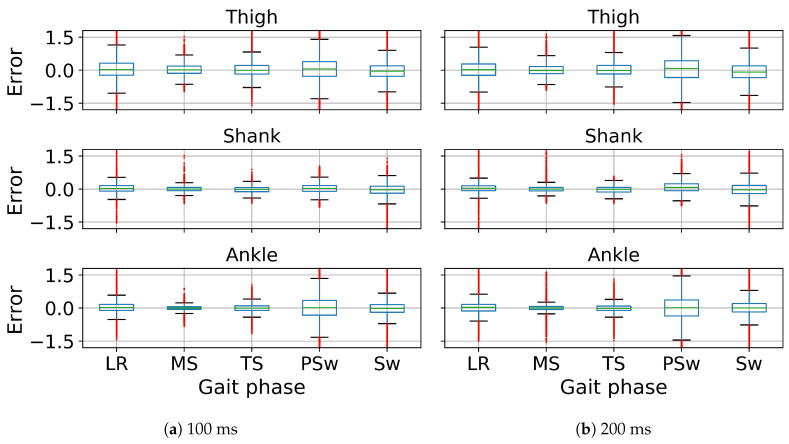
Error of angular velocities of ipsilateral thigh, shank and foot in 100 ms (**a**) and 200 ms (**b**) ahead prediction with respect to gait phases in inter-subject implementation. All angular velocity values shown are normalized to have zero mean and unit variance. The line inside the box represents the median error. The box represents the middle 50% of the error. The whiskers represent the ranges for the bottom 25% and the top 25% of the error, excluding outliers. The red tails indicate the outliers.

In gait trajectory prediction, the LSTM was still able to predict trajectories from an unseen subject based on the other subjects’ trajectories in the 100 and 200 ms ahead predictions (Figure 9). The shank segment was again predicted with the highest correlation and SNR as well as the least MAE and RMSE (Figure 10, Table 1). There is again a strong relationship between the predicted and true trajectory in inter-subject implementation with all $R^{2}$ larger than 0.8 (illustrated for one subject in Figure 10), though the variance is somewhat larger than that in intra-subject implementation for the same subject (Figure 6). The errors of the predicted trajectories for unseen subjects were also close to zero, and the smallest variance occurred in MS and TS (Figure 11). All trajectory prediction errors are generally larger in the inter-subject than in the intra-subject implementation (Figure 7 and Figure 11).

## 4. Discussion

The main finding of our study is that the proposed LSTM network can reliably predict the trajectory of the thigh, shank and foot segments as well as the gait phases for up to the subsequent 10 time frames, equivalent in this case to 200 ms ahead. In both intra- and inter-subject implementations, the high correlations between true and predicted trajectories as well as the SNR values all greater than zero are particularly encouraging. Our results show higher correlation and lower noise compared to the result achieved by Presacco et al. [37]. The main novelty to our approach is the designated loss function that incorporates a discount factor to model the uncertainty of future prediction. Empirical events are in general easier to predict in the immediate future than in the more distant future. The discount factor can be seen as a measure to indicate the difficulty of prediction far in the future. Most neural networks including the LSTM are usually trained by gradient descent to determine optimum model parameters that can minimize the cost function. However, gradient descent would not always find a clean solution due to the non-linearity of the neural network, which entails the loss function to become non-convex. This means that the neural network might be trained to reach a low error of the cost function with gradient decent, but would be no guarantee of a global convergence for such non-convex functions [38].

Since the gait trajectories are periodic with a repetitive nature and a temporal pattern, the model performance for all multiple time frames can be increased when the model is forced to minimize the error in the most immediate time frames with a discount factor. If the discount factor is too small, however, the total loss will be reduced quickly even if the weights are not adequately updated to reduce the error between the predicted trajectory and the true trajectory. If the discount factor is instead too large, the model will place equal weight on predicting the most immediate and the more distant time frames. The discount factor was designed to more accurately predict the near future—100 and 200 milliseconds at our sampling frequency—while maintaining an overall good performance during the entire prediction horizon. The optimum discount factor for gait trajectory prediction was determined to be 0.8, as it resulted in the lowest RMSE for all gait trajectories (see Figure 2b). On the other hand, the discount factor affects the model in gait phase prediction differently. The highest prediction accuracy is attained with a discount factor of 1.0, which means that the loss is not weighted for different time frames (Figure 2a). The reason may be that gait phase prediction is essentially a classification problem, which uses a softmax function to output the probability distribution of five gait phases. Softmax is exponential and enlarges differences between classes—it pushes the most likely class closer to one and the most unlikely class closer to zero. Although the discount factor affects the probability distribution, it does not alter the final output of gait phases to a great degree. For instance, if the probability of five phases in a time frame is {0.1,0.15,0.15,0.2,0.4} with no discount, corresponding to LR, MS, TS, PSw, Sw, respectively, the predicted gait phase is Sw. If a discount factor other than one is applied, say the probability of the five gait phases becomes {0.1,0.15,0.15,0.1,0.5}, the predicted gait phase will still be Sw.

As the average gait cycle duration in adults ranges from 0.98 to 1.07 s [39], segment trajectory prediction for the subsequent 200 ms, which corresponds to nearly 20% of a normal gait cycle, can be used in a feed-forward exoskeleton controller that can react in advance to compensate for any potential controller time delays [3]. Prediction error MAE gradually increased for each of the five time frames (a 100 ms duration), indicating better prediction in the most immediate time frames as a result of the weighted loss function (Figure 3). Wang et al. [40] used a cost-minimizing control strategy computed for a discrete time period in the future for the LOPES [41] exoskeleton. Their controller minimized the difference between the true and predicted gait trajectories for the whole prediction horizon to produce joint torque for the exoskeleton for the next time step. Our work of multiple time steps prediction of gait trajectories can inherently fit into this control framework to provide an adaptive gait trajectory since the gait trajectory presented in our work need not be normalized to the gait cycle.

In terms of gait phase prediction, the proposed LSTM approach can accurately predict the five gait phases, particularly the swing phase, and false predictions mainly occur in the adjacent gait phases. Our finding supports designs of different control modes for stance and swing individually as assistive devices tend to provide more force to support and propel the wearer during stance than swing [42]. Sharbafi et al. [43] developed a control algorithm of an exoskeleton with one biarticular actuator upon a reflex-based human walking model that employs the leg force to adjust hip compliance. In a reflex-based model, gait phase prediction is a vital part to the control structure as it switches each leg to stance or swing phase between the different reflexes. Our approach of gait phase prediction is applicable in this type of model.

As expected, the intra-subject implementation shows better performance in both gait trajectory and gait phase prediction than inter-subject implementation, which suggests that subject-specific training is preferable if the gait trajectory and gait phase are to be used for an exoskeleton framework. Although a generic model can reduce the time required in the training process, some studies have shown that a generic model may not be suitable for specific individuals, and that subject-specific musculoskeletal geometry is essential for high prediction accuracy [44,45]. One advantage of our approach is that no anthropometric geometry measurements are needed in either implementation. In addition, the labeling process is fully automated, so that no intervention from a human operator is required once the IMUs and foot switches are attached. The mean error of the predicted trajectory in both implementations was close to zero, which suggests that the model has little or no systematic error. While all predicted segment trajectories were in general close to their true trajectories, the shank trajectory was overall best predicted, and particularly high during the mid- and terminal stance. This can inspire development of an exoskeleton control framework that emphasizes following the trajectory of the shank to achieve smoother synchronization.

There are some limitations to the current study. The experiment is limited to 12 healthy young subjects and confined only to treadmill walking. In the future, experiments with more participants and populations with variations in gait function, such as older people and patients with walking disabilities, should be included to validate the applicability of this approach in a wider context. Walking in different conditions such as uphill or downhill or on uneven surfaces can also test the robustness of this methodology. Furthermore, the proposed LSTM network was of standard LSTM architecture but designed to perform multi-time step prediction with the novelty of an incorporated loss function wherein the errors and accuracy are computed in the entire predicting horizon. In future studies, it would be interesting to compare the multiple-time step prediction with single time step prediction in an optimal recurrent network architecture, as per for example, Jozefowicz et al. [46], who achieved this by adjusting different input, output and forget gates. Furthermore, any potential issue that may result from incorrectly predicted gait phases might cause an unstable response in exoskeleton control, but can be addressed with an impedance-based controller that only changes the impedance of the exoskeleton at the transition between gait phases [47]. In addition, the performance of the LSTM network in the inter-subject implementation may be further improved by extracting more representative features out of the 63 signals, such as the mean, maximum, and minimum values within a time window. Finally, it is worth pointing out that we tested the LSTM network’s ability to reliably predict phases for discrete numbers of time frames. We down-sampled our collected sensor data from 2000 to 50 Hz to attain 20 ms time frames, and could thus achieve prediction for 200 ms ahead. We speculate that prediction for 200 ms could be achieved with this LSTM network through a greater number of smaller time steps, but we have not verified this speculation.

## 5. Conclusions

The proposed LSTM network with a weighted discount loss function can overall predict gait trajectory (angular velocities of thigh, shank and foot segments) and gait phases (loading response, midstance, terminal stance, pre-swing and swing) for multiple time frames equivalent to the next 200 ms. While the model is designed to more accurately predict the gait trajectory in the most immediate future than in the more distant future, the average prediction performance within the entire prediction range (100 or 200 ms) can be improved with a suitable discount factor. The discount factor does not, however, affect gait phase prediction, which is accurately predicted using the proposed LSTM network. The ability of the LSTM network to predict future gait trajectories and gait phases can be applied in designing exoskeleton controllers that can better compensate for system delays to smooth the transition between gait phases.

## Figures and Tables

**Table 1:** Model performance for z-score normalized angular velocities of ipsilateral thigh, shank and foot segments for 100 and 200 ms ahead predictions of intra-subject and inter-subject implementations, shown as mean error, MAE, RMSE, R and signal to noise ratio (SNR). The sign of the error indicates the relative direction of the error; a negative error means that the average true value is lower than the average predicted value, and an error of zero indicates that the average predicted value was the same as the average true value.

Prediction	Segment	Intra-Subject/Inter-Subject				
		Error	MAE	RMSE	R	SNR
100 ms	Thigh	0.000/0.011	0.127/0.308	0.203/0.428	0.98/0.91	14.12/7.85
	Shank	0.003/0.004	0.063/0.187	0.093/0.257	0.99/0.97	20.73/13.27
	Foot	0.002/−0.002	0.107/0.266	0.183/0.425	0.98/0.93	14.74/8.93
200 ms	Thigh	0.004/−0.004	0.148/0.300	0.231/0.423	0.97/0.91	13.03/7.56
	Shank	0.001/0.007	0.073/0.208	0.106/0.304	0.99/0.96	19.53/11.98
	Foot	0.003/0.005	0.118/0.299	0.203/0.487	0.98/0.91	13.82/8.04

## Abbreviations

The following abbreviations are used in this manuscript:

LSTM	Long-short term memory
LLEs	Lower limb exoskeletons
IMU	Inertial measurement unit
LR	Loading response
MS	Midstance
TS	Terminal stance
PSw	Pre-swing
Sw	Swing
Adam	Adaptive moment estimation
MAE	Mean absolute error
RMSE	Root mean squared error
SNR	Signal to noise ratio
